# Is Sotolon Relevant to the Aroma of Madeira Wine Blends?

**DOI:** 10.3390/biom9110720

**Published:** 2019-11-09

**Authors:** João M. Gaspar, Ana I. Freitas, Qianzhu Zhao, João M. Leça, Vanda Pereira, José C. Marques

**Affiliations:** 1 Faculty of Exact Sciences and Engineering, University of Madeira, Campus da Penteada, 9020-105 Funchal, Portugal; 2 Institute of Nanostructures, Nanomodelling and Nanofabrication (I3N), University of Aveiro, 3810-193 Aveiro, Portugal

**Keywords:** 3-Hydroxy-4,5-dimethyl-2(5*H*)-furanone, wine aroma, fortified wine, odor thresholds, odor activity value, sensory analysis

## Abstract

Madeira wine (MW) oxidative aging results in the formation of several key aromas. Little is still known about their odor relevance to the aroma of the most commercialized MWs. This report presents an in-depth study of the odor impact of sotolon in MW blends. First, its odor perception was estimated in MWs according to ASTM E679, testing different 3-year-old (3-yo) commercial blends. The odor relevance of sotolon in the aroma of 3-, 5-, and 10-yo commercial blends (89 MWs) was then appraised by calculating its Odor Activity Value (OAV), after determining its content by RP-HPLC-MS/MS. The sotolon odor perception in MW was as low as 23 µg/L, although it was found that little differences in the wine matrix influenced its perception. OAVs varied between 0.1 and 22, increasing with the blend age. Considering that 16% of the OAVs are higher than 10 (mostly ≥ 10-yo), sotolon was found to be a key contributor to the overall aroma MW blends.

## 1. Introduction

Madeira is a world-renowned fortified wine (17–22% alcohol by volume, ABV) with a great historical and economic value. These wines are produced in different styles, with concentrations of unfermented sugars up to more than 96.1 g/L [1]. Madeira wine (MW) oxidative aging takes place at up to about 30–45 °C and is thought to contribute to its longevity and robustness, making these wines unique. More information about its processing can be found elsewhere [2,3]. After aging, MWs can be bottled according to two categories: those with a generic age, known as blends (3-, 5-, 10-, 15-, and ≥ 20-year-old (20-yo) MW) and those produced from a single harvest and grape variety, *Colheitas* or *Frasqueiras*. Most of the bottled MW is commercialized as 3-, 5-, and 10-yo blends [4]. The price for 0.75 L MW bottles ranges from about 5–10 € for 3-yo, 9–16 € for 5-yo, and 19–40 € for 10-yo [5,6].

The oxidative nature of MW aging promotes the development of intense and complex aromas [2,7,8,9]. These fortified wines are rich in aging aromas such as dried fruit, nutty, musty, baked, oak, mushroom, and brown sugar, often overlapping the existing varietal aromas. The overall aroma composition of four 10-yo MW blends was previously studied by Campo et al. [9], which found that these wines were rich in wood extractable compounds such as *(Z)-*whiskylactone, volatile phenols, and important odor active compounds, such as phenylacetaldehyde and sotolon. Particularly, sotolon (3-hydroxy-4,5-dimethyl-2(5*H*)-furanone) was pointed out as having a high odorant impact in these wines and has been associated with their characteristic nutty aroma. This chiral lactone is present in many foodstuffs and wines, to which it can impart a nutty/caramel/curry/rancid odor, depending on its concentration and enantiomeric distribution [10,11]. Sotolon is perhaps best known for being an off-flavor of young dry white table wines and its presence has been related to premature oxidative aging phenomena [10,12,13]. However, in fortified wines like Sherry, Port, and Madeira, sotolon is considered a key-aroma and has been quantified in concentrations as high as 500 [14], 958 [15], and 2000 µg/L [16], respectively. Sotolon has also been established as an important aging marker of MW [16,17]. Câmara et al. [16] evaluated the influence of the aging period and sugar contents on the levels of sotolon and have found that it was strongly correlated with aging time and sugar derivatives such as  furfural, 5-methylfurfural, 5-hydroxymethylfurfural, and 5-ethoxymethylfurfural. Pereira et al. [18] reported that the origin of sotolon in sweet fortified wine can be mostly associated with sugar degradation mechanisms.

Some advances in the MW winemaking process have been introduced in recent years and can now be reflected in the wine aroma. Considering that the quality of fortified wines is usually associated with the aroma contribution of sotolon, it becomes important to appraise its impact on MWs produced nowadays. After the preliminary attempt to estimate its odor threshold in sweet MW [19], this work provides a detailed study about the impact of sotolon on the aroma of commercially available MWs, covering a wide sample set composed of 89 blends of 3-, 5-, and 10-yo of different styles and varieties from four different MW producers, and measuring the corresponding odor activity values (OAVs). The perception threshold of this odorant was estimated in the MW through the sensory evaluation of 3-yo blends by a selected panel. The matrix effect was appraised.

## 2. Materials and Methods

### 2.1. Chemicals

All chemicals used had a purity grade higher than 97%. Ethyl acetate was purchased from Fisher Scientific (Leicestershire, UK). Formic acid, tartaric acid, and sodium hydroxide were obtained from Panreac (Barcelona, Spain) and HPLC-grade methanol was from Chem-Lab (Zedelgem, Belgium). Sotolon food grade standard and absolute ethanol were purchased from Sigma-Aldrich (St. Louis, MA, USA). Ultra-pure water (type 1) was obtained from a Simplicity^®^ UV ultra-pure water apparatus from Millipore (Milford, MA, USA).

### 2.2. Sotolon Odor Threshold

#### 2.2.1. Panelists

Six panelists (five females and one male, with ages ranging between 30 and 46 years old) from 22 non-trained individuals, recruited from the University of Madeira, were selected, based on their acuity and sensitivity to the olfactory stimulus. The selection was based on the overall preliminary tasting performances during a previously reported study [19], established by the percentage of correct responses (at least 50%) and by individual best estimate odor threshold (BET) scores (panelist with individual BETs much higher than the previously found for MW in [19] were not selected), as shown in Table S1. Although the selected panelists were not frequent drinkers of Madeira or other fortified wines, all had prior experience in MW tastings and sensory analysis evaluations. These panelists were familiarized with the sotolon stimulus by repeat exposure in previous sensory trials but were not specifically trained for this study. The participants also signed an informed consent form and completed a simple questionnaire.

#### 2.2.2. Samples

For the sensory tests, 4 MW blends with vestigial contents of sotolon were selected. These included 2 commercially available 3-yo wines of 2 different styles (dry and sweet) from 2 local producers. Table 1 shows the average values of the basic oenological parameters of these wines obtained on a TDI (Barcelona, Spain) Bacchus 3 Multispec analyzer, equipped with an iD1 transmission accessory. The equipment includes a Nicolet iS5 rapid-scanning Fourier-transform infrared spectrophotometer from Thermo Scientific (spectral range between 7800–350 cm^−1^), with CaF_2_ windows, a Czerny-Turner UV-Vis spectrophotometer (250–600 nm) fitted with 0.2 mm flow cells, and a temperature-regulated auto-sampler. Calibrations for the determination of alcoholic strength, density, volatile acidity, titratable acidity, and pH were previously established, following the OIV reference methods [20]: OIV-MA-AS312-01A:R2016, OIV-MA-AS2-01A:R2012, OIV-MA-AS313-02:R2015, OIV-MA-AS313-01:R2015, and OIV-MA-AS313-15:R2011, respectively. An internal procedure based on the Lane–Eynon method [21] was used to calibrate the residual sugars.

Standard stock (3.96 g/L) and working (100 mg/L) solutions of sotolon in ethanol and in synthetic wine (18% ethanol; 6 g/L of tartaric acid; pH adjusted to 3.5 with 1 M sodium hydroxide solution) were first prepared, respectively. The working solution of sotolon was used to spike the wines used for the sensory tests. The selection of the concentration range to be evaluated in the sensory study had in consideration the results of preliminary trials (Table S1). A 2.5-fold ascending concentration series was set, diluting the working solution into each wine to obtain concentrations of 16, 40, 100, 250, and 625 µg/L of sotolon. Wine solutions were prepared the day before each sensory session and left overnight at ambient temperature to equilibrate.

#### 2.2.3. Sensory Tests

The ascending forced choice method of limits described by the American Society for Testing and Materials (standard practice ASTM E679) was followed to determine the orthonasal best estimate odor threshold (BET) in MW [22]. The evaluation was performed using the previously described 3-yo MW blends. This standard practice is a fast and reliable method for determining the detection threshold of a stimulus with only 3-alternative forced choice (3-AFC) presentations. Each presentation set comprised a triad of ISO tasting glasses, 1 filled with 30 mL of spiked wine (test sample) and 2 others filled with non-spiked wines (blank samples). All wine glasses were coded with a 3-digit random number, covered with plastic petri-dishes and randomly arranged. The tasting sets were prepared 1 h prior to the sensory evaluations. Panelists were instructed to smell each sample and choose the different sample from each triad, starting from the lowest concentration scale-step. Panelists were asked to make a guess in case of uncertainty. The responses were registered in a paper ballot by circling the selected coded sample. Panelists were also asked not to eat, drink, or smoke during the 30 min prior to the tasting session. Each sensory study was composed of duplicate sessions per day, totaling 55 3-AFC presentations per day per wine. All evaluation tests were conducted in a temperature-controlled room free of odors, noise, and other major distractions, at the University of Madeira. Individual BETs were determined by taking the geometric mean of the highest incorrect concentration and the next higher correct concentration scale-step, provided that in the following scale-steps the panelist made consistently correct selections. In case of an incorrect response at the highest concentration available, the individual BET was obtained by taking the geometric mean of that concentration and the next hypothetical higher concentration. Similarly, in the case of a complete run of correct selections, the BET was calculated by taking the geometric mean of the lowest concentration and the next hypothetical lower concentration. The arithmetic mean of replicate sessions was calculated as the final individual BET, and the group BET was calculated as the geometric mean of all final individual BETs.

### 2.3. Sotolon Odor Impact in MW Blends

The odor relevance of sotolon to the aroma of 3-, 5-, and 10-yo blends was appraised by calculating the OAV of sotolon in each wine. OAVs were obtained by dividing the quantified concentration of sotolon by the estimated odor threshold.

#### 2.3.1. Wines

Eighty-nine MW blends were sampled in 2017 from 4 different producers, comprising all sweetness styles (dry, medium dry, medium sweet and sweet). Table 2 describes in more detail the number of samples analyzed.

#### 2.3.2. Sotolon Extraction and Analysis

The concentrations of sotolon in wines were determined according to a previously published method [23], through a miniaturized liquid–liquid extraction procedure followed by RP-LC-MS/MS analysis. Briefly, in 50 mL PTFE centrifuge tubes, 8 mL of ethyl acetate was added to 15 mL of the sample. The mixture was vortexed for 5 min and then centrifuged for 10 min at 4400 rpm. After separation, the upper phase was collected and evaporated under a slow nitrogen flow. The residue was dissolved in 0.1% formic acid up to a final volume of 1 mL and filtered through Chromafil Xtra PTFE 0.20 µm syringe filters (Macherey-Nagel, Düren, Germany). Five microliters of the sample extract were then injected into a Nexera X2 LC system (Shimadzu Corporation, Kyoto, Japan) equipped with SIL-30AC autosampler, binary LC-30AD pumps, DGU-20 A5 degassing unit, CTO-20A column oven, and LCMS 8040 triple-quadruple mass spectrometer equipped with an ESI ionization module. Separation was achieved using a Kinetex reversed-phase C18 column (150 × 2.1 mm, 2.6 μm, 100 Å) from Phenomenex (Torrance, CA, USA), at 40 °C, and using a gradient elution: 5% solution A maintained for 4 min, increased to 30% in 2 min, increased to 100% in 1 min, decreased to 5% in 3 min, and held at 5% for 5 min. Solution A was methanol and solution B was 0.1% formic acid in water, set to a 0.4 mL/min flow rate, for a total run time of 15 min. Both solutions were previously filtered through a hydrophilic PP membrane filter from Pall Corporation (Ann Arbor, MI, USA), with a 0.2 µm pore size. The MS detector was set to acquire between 0.5 and 9.0 min, in positive ion mode using multiple reaction-monitoring (MRM) mode. Settings were as follows: desolvation line was kept at 250 °C and block heater at 400 °C; nebulizing gas flow was set at 2.5 L/min and the drying gas flow at 17.5 L/min. The transitions 129.1 m/z ⟶ 83.0 m/z and 129.1 m/z ⟶ 55.1 m/z were monitored for identification and the transition 129.1 m/z ⟶ 55.1 m/z was used for quantification purposes. A chromatogram with sotolon retention time and MS/MS spectrum can be found in the supplementary material (Figure S1). All extracts were injected in duplicate and data was acquired and processed using the Labsolutions 5.7 software from the Shimadzu Corporation. Each wine sample was analyzed in duplicate.

### 2.4. Statistics

Significant differences were evaluated by the analysis of variance (one-way ANOVA with the Holm-Sidak method) using the Minitab 17 statistical software (Minitab, LLC, State College, PA, USA). Box Plot data analysis was performed in the Excel of Microsof Office 365 ProPlus.

## 3. Results and Discussion

### 3.1. Odor Threshold Determinations

After 55 3-AFC presentations per wine, the odor thresholds were calculated as described before. Table 3 contains the group BETs obtained for the different 3-yo MWs chosen for the odor threshold evaluation. The BETs ranged from 23.3 to 68.7 µg/L.

The wines from producer A had lower BET values than those found for producer B. Like all wine producers, MW producers seek to maintain their identity in the sensory characteristics of their wines, and little differences in wine matrices are found (Table 1). Matrix effects are known to influence the perceptiveness of a specific stimulus in alcoholic beverages [24,25,26]. It is recognized that matrix components interact with volatile flavors, namely aromas [27]. However, their retention and release are a complex matter. The flavor partitioning between liquid and gas can be influenced by the presence of these components that affect the chemical activity of the flavor. For example, ethanol, a polar volatile component of alcoholic beverages, is fully miscible with water, which increases the solubility of hydrophobic aromas and therefore enhances their retention. Proteins can also retain these volatiles, while sugars can influence the water activity and facilitate aroma release. Thus, a more appropriate measurement of the odor threshold of sotolon can be achieved using the MW matrix itself, since other volatile and non-volatile constituents, such as ethanol and/or sugars, can eventually affect the sotolon volatility and release. This fact can explain the observed variability of the determined threshold values for the four MWs studied. The sotolon BET obtained for the sweet-style from producer A was higher than the corresponding dry-style, while the opposite was observed for those from producer B. Despite the differences found between the BETs of different wine styles from the same producer, these are not as relevant as those found between producers. These differences seem to be more closely related to overall wine composition than with wine sugar content by itself (Table 1). This suggests that sugar content and wine style were not a key factor that influences sotolon perceptibility in these wines. It is also noteworthy that natural variability of the sensory tests may also account for such differences [22,28].

In a recent study, regarding the sotolon odor threshold in MW, Gaspar et al. (2018) [19] found a group BET of 112 µg/L for a sweet-type 3-yo blend, obtained from sensory evaluations of 19 non-trained and non-expert panelists. In this specific case, the acuity of the selected panel to better recognize sotolon is the most probable reason to explain the discrepancy in the threshold values obtained in both studies. Besides panel selection and individual panelist acuity, the familiarization gained from repeated exposure to the stimulus is expected to improve the overall panel discrimination ability [28,29], which can also justify the odor threshold decrease observed in the current study. The calculated BETs are now closer to the odor threshold previously estimated for Port wine (19 µg/L) [15], a fortified wine with similar organoleptic characteristics [1]. The effect of the matrix complexity can also be observed when comparing the sotolon threshold values with those reported by Campo et al. [9] in a model wine solution: an orthonasal odor detection threshold of 9 µg/L for sotolon in a 10% (*v*/*v*) water/ethanol mixture containing 5 g/L of tartaric acid at pH 3.2.

Thus, the group BET of 23.3 µg/L obtained for the dry-style wine from producer A can be considered as the sotolon odor threshold in MW.

### 3.2. Sotolon Odor Impact in MW Blends

**Figure 1:**
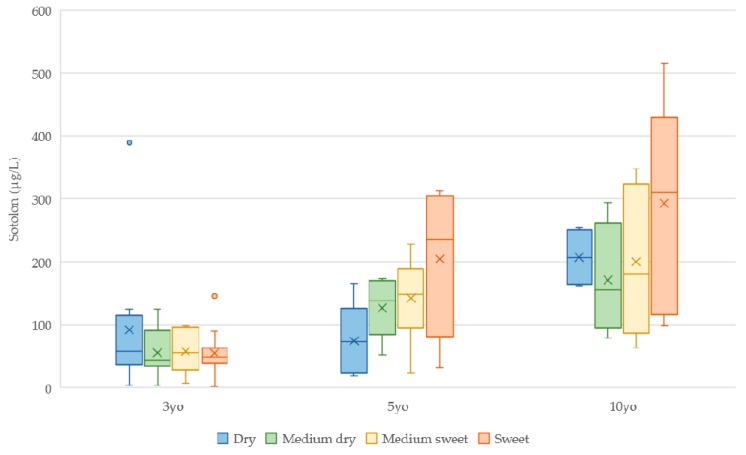
Box plot of sotolon contents (in µg/L) in 3-, 5- and 10-year-old MW blends.

The odor relevance of sotolon on the aroma of 3-, 5- and 10-yo commercial MW blends (89 samples) was also appraised by calculating the OAVs, after determining the sotolon content in each sample by RP-HPLC-MS/MS, as previously described. Sotolon contents ranged from 2.0 ± 0.9 µg/L to 516 ± 17 µg/L (Table S2). Figure 1 shows the box plot of sotolon concentrations in the studied wines according to age and style.

As expected, the sotolon contents of each style increase with the age of the blend. It is also noticeable that there is data dispersion, mostly in older wines, which might be because MW blends are a mixture of different wines with different ages. Considering that, the relation between sotolon concentration and sugar content in this case is not clear, but a general increase with age is noticeable, confirming that sotolon can be considered as a wine aging marker.

**Figure 2:**
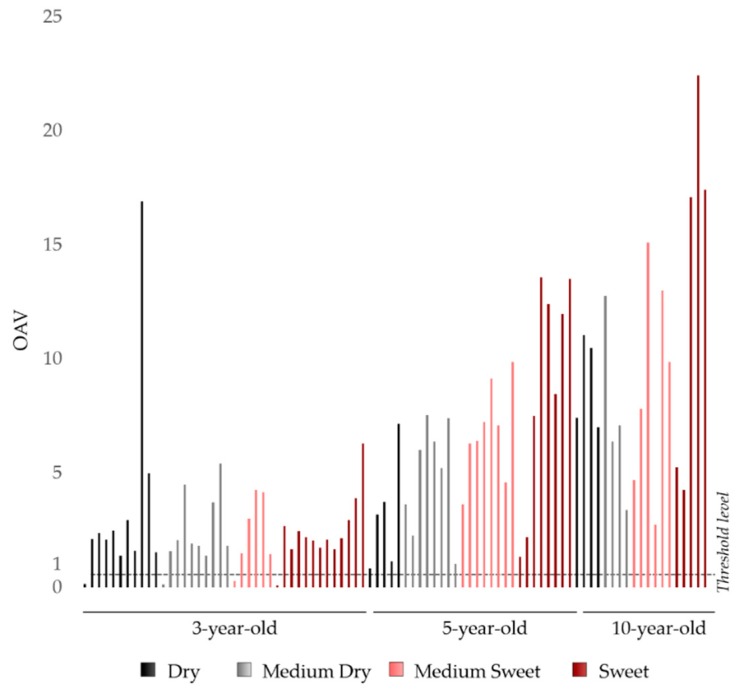
Odor activity values (OAVs) of sotolon determined in 3-, 5-, and 10-year-old MW blends.

The OAVs were calculated as the ratio between the quantified sotolon concentration and the lowest BET value previously determined (23.3 µg/L). Figure 2 presents the OAVs found for the studied wines. If an aroma compound is present in concentrations at or above its odor threshold (OAV ≥ 1), it is generally recognized that it can be perceived and the larger its magnitude is, the more likely it is to have a greater contribute in the overall aroma of a wine.

OAVs in this sample set ranged from 0.1 to 22, with an OAV mean value of 2.8 for 3-yo, 6.3 for 5-yo, and 9.8 for 10-yo wines. Ninety-four percent of MW blends presented OAVs ≥ 1, which shows that sotolon is perceptible in most wines. OAVs < 1 were only observed in five wines, and among these, four were 3-yo blends. Fourteen MWs (16%) had OAVs ≥ 10, mostly 10-yo blends (Figure 2). These results confirm that the odor relevance of sotolon is higher in older wines. The wine age is known to have a correlation with the sotolon content of MWs [16]. It has also been suggested that sotolon is correlated to sugar content, and sugar degradation mechanisms may play a role in its formation in this kind of beverage [16,18]. This relationship is not so clear in this study, since the wines analyzed are commercial blends, i.e., mixtures of different wines, reflecting the aging practices of each producer. However, higher OAV values (5- and 10-yo blends) were found in the sweet-style MW blends (Figure 2). Nevertheless, the current study shows that sotolon has a relevant odor impact in blended wines of different ages and should be taken into consideration before preparation.

The first OAV data for sotolon in 10-yo MW blends (4 samples) was reported by Campo et al. [9], with OAVs ranging between 1.6 and 2.1. The average OAV obtained for the 10-yo MW commercial samples in the current study (n = 19) is significantly higher (9.8), reflecting the changes that might be introduced. Thus, this result suggests that more elevated of sotolon may be found in the currently commercialized MW blends.

## 4. Conclusions

This study shows that sotolon has an important contribution to the aroma of commercially available MW blends, particularly in those of ages higher than 10 years old. Sotolon has a lower odor impact on the aroma of younger wines (3-yo), since lower concentrations are found, some very low (OAV < 1). This can reveal that small differences in the winemaking and/or aging processes might have been applied by the different producers. The odor impact of sotolon in these fortified wines may have increased in recent years, considering that OAVs increased significantly in the current MW blends, namely in consequence of undergoing studies. Changes in the winemaking/aging process will continue to be studied to potentiate the formation of sotolon, mostly in young wines, and the use of new approaches can further decode in more detail the aroma signature of MW.

## Figures and Tables

**Table 1:** Oenological parameters of commercial 3-year-old Madeira wines (MWs) selected for the sotolon sensory study.

	Producer A		Producer B	
	Dry MW	Sweet MW	Dry MW	Sweet MW
alcohol (% ABV)	18.03 ± 0.01 ^a^	18.53 ± 0.02 ^b^	19.23 ± 0.03 ^c^	19.28 ± 0.03 ^c^
density (g/mL)	1.0033± 0.0001 ^a^	1.0263 ± 0.0002 ^b^	1.0049 ± 0.0002 ^c^	1.0274 ± 0.0003 ^d^
volatile acidity (g/L)	0.39 ± 0.01 ^a^	0.40 ± 0.00 ^a^	0.44 ± 0.03 ^a,b^	0.49 ± 0.04 ^b^
titratable acidity (g/L)	4.46 ± 0.06 ^a^	4.92 ± 0.01 ^b^	5.1 ± 0.1 ^b^	4.95 ± 0.06 ^b^
pH	3.52 ± 0.01 ^a,b^	3.51 ± 0.01 ^a^	3.51 ± 0.01 ^a^	3.54 ± 0.02 ^b^
residual sugars (g/L)	52.1 ± 0.8 ^a^	112.9 ± 0.5 ^b^	63 ± 1 ^c^	120 ± 1 ^d^

The values were obtained from triplicate analysis and are expressed as mean ± standard deviation. Different letters denote statistically significant differences (*p* < 0.05) by Holm-Sidak test.

**Table 2:** Number of samples analyzed to assess the odor relevance of sotolon in MW blends according to age and style.

Age	Style	No. Samples
3-year-old	dry	11
	medium dry	10
	medium sweet	6
	sweet	14
5-year-old	dry	5
	medium dry	7
	medium sweet	9
	sweet	8
10-year-old	dry	4
	medium dry	4
	medium sweet	5
	sweet	6

**Table 3:** Group best estimate odor thresholds (BETs) obtained for the orthonasal odor threshold of sotolon in 3-year-old MWs.

	Producer A		Producer B	
	Dry MW	Sweet MW	Dry MW	Sweet MW
group BET (µg/L) ^a^	23.3	35.3	68.7	41.7
log_10_	1.4	1.5	1.8	1.6
log_10_ standard deviation	0.4	0.6	0.6	0.7

^a^ Group BETs calculated as the geometric means of the individual BETs from six panelists during duplicate sessions.

## Supplementary Materials

The supplementary materials are available online at https://www.mdpi.com/2218-273X/9/11/720/s1.
