# Supplementary material for: Is Sotolon Relevant to the Aroma of Madeira Wine Blends?
Source: Biomolecules. 2019 Nov 9;9(11):720. doi: 10.3390/biom9110720 (PMC6920768; doi:10.3390/biom9110720)
Supplement: Supplementary file 1 [file biomolecules-09-00720-s001.pdf]

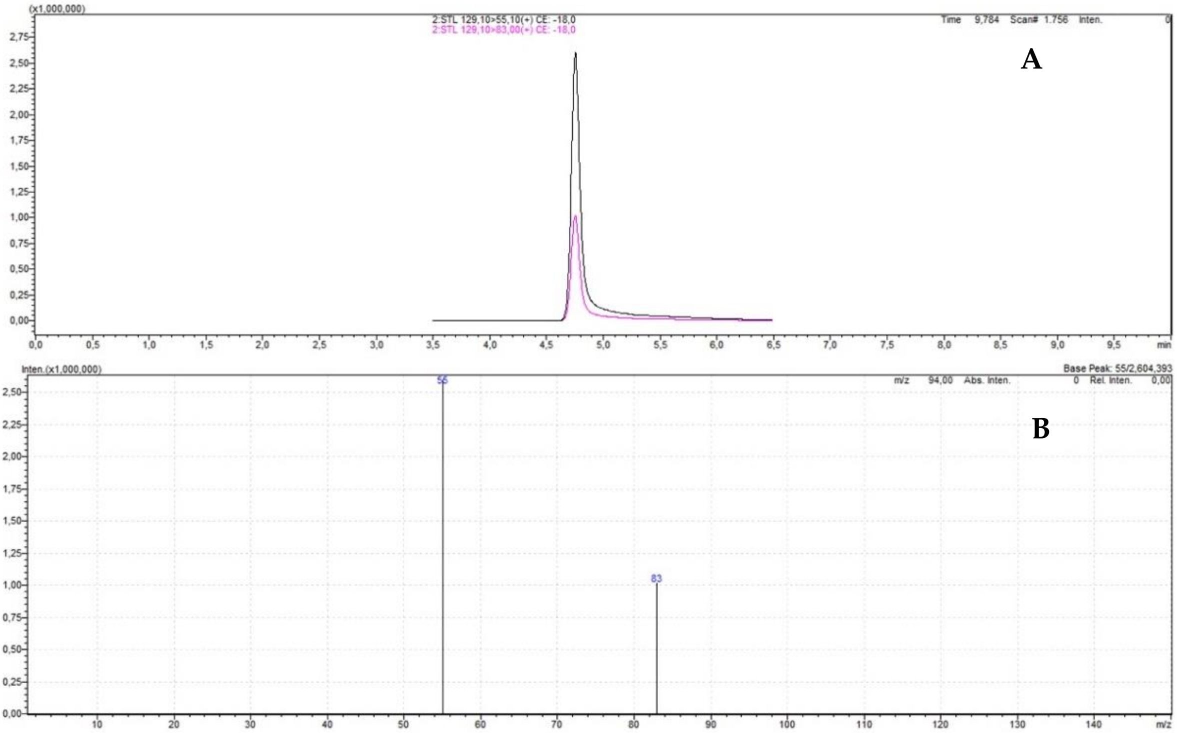

Figure S1. LC-MS chromatogram (A) of a MW wine extract analysis with the corresponding sotolon MS/MS spectrum (B).

Table S1. Performance of the 22 individuals in preliminary trials: percentages of correct responses; number of 3-AFC tests completed; and individual best estimate thresholds (BETs). In bold are highlighted the individuals selected for the panel.

Individuals	% correct responses	# of 3-AFC	BET
P1	30	10	170
P2	30	10	170
P3	60	10	226
**P4**	**52**	**36**	**125**
**P5**	**58**	**22**	**71**
P6	38	11	170
**P7**	**67**	**36**	**93**
P8	62	35	141
P9	56	36	202
P10	38	16	264
**P11**	**77**	**27**	**80**
P12	35	36	921
P13	48	30	543
**P14**	**70**	**41**	**67**
P15	33	17	170
P16	42	12	283
**P17**[a]	**67**	**6**	**57**
P18	67	6	453
P19	59	19	937
P20	43	7	1358
**P21**	**86**	**7**	**85**
P22	71	7	170

[a] Panelist P17 would be selected but was not available during the current study.

Table S2. Sotolon concentration data of 3-, 5- and 10-year-old MW blends (mean ± standard deviation).

Category	Style	Concentration (µg/L)
3 YO	Dry 1	82.2±4.0
	Dry 2	3.6±1.0
	Dry 3	48.6±2.3
	Dry 4	54.8±2.5
	Dry 5	48.4±2.7
	Dry 6	57.5±2.3
	Dry 7	32.1±4.4
	Dry 8	68.3±1.2
	Dry 9	37.0±0.4
	Dry 10	389.2±12.3
	Dry 11	115.5±9.8
	Medium Dry 1	35.5±4.7
	Medium Dry 2	3.3±0.3
	Medium Dry 3	36.8±0.7
	Medium Dry 4	48.0±1.4
	Medium Dry 5	103.5±4.1
	Medium Dry 6	44.2±4.7
	Medium Dry 7	42.3±1.7
	Medium Dry 8	32.2±3.0
	Medium Dry 9	86.0±0.5
	Medium Dry 10	125.2±3.9
	Medium Sweet 1	42.1±6.8
	Medium Sweet 2	6.9±0.6
	Medium Sweet 3	34.5±1.6
	Medium Sweet 4	69.7±0.9
	Medium Sweet 5	98.8±1.4
	Medium Sweet 6	96.1±0.7
	Sweet 1	33.6±9.2
	Sweet 2	2.0±0.9
	Sweet 3	61.9±10.1
	Sweet 4	38.9±0.7
	Sweet 5	57.1±5.1
	Sweet 6	50.8±3.8
	Sweet 7	47.3±2.3
	Sweet 8	40.4±2.9
	Sweet 9	48.4±2.6
	Sweet 10	38.8±1.3
	Sweet 11	49.8±3.2
	Sweet 12	68.3±1.4
	Sweet 13	89.9±7.1
	Sweet 14	145.5±7.6
5 YO	Dry 1	19.4±2.8
	Dry 2	73.6±7.2
	Dry 3	86.2±10.3
	Dry 4	26.7±2.0
	Dry 5	165.3±7.1
	Medium Dry 1	84.0±1.7
	Medium Dry 2	52.4±4.6
	Medium Dry 3	138.6±4.5
	Medium Dry 4	173.5±8.0
	Medium Dry 5	147.2±1.6
	Medium Dry 6	120.2±4.0
	Medium Dry 7	170.7±4.4
	Medium Sweet 1	23.9±0.4
	Medium Sweet 2	84.0±7.8
	Medium Sweet 3	145.4±8.8
	Medium Sweet 4	147.9±9.6
	Medium Sweet 5	167.0±11.0
	Medium Sweet 6	210.9±4.3
	Medium Sweet 7	163.7±2.1
	Medium Sweet 8	106.5±14.2
	Medium Sweet 9	227.7±14.2
	Sweet 1	31.1±3.6
	Sweet 2	50.7±3.5
	Sweet 3	173.4±11.7
	Sweet 4	313.0±4.4
	Sweet 5	286.0±6.0
	Sweet 6	195.1±14.8
	Sweet 7	275.6±5.7
	Sweet 8	311.4±9.1
10 YO	Dry 1	171.0±16.0
	Dry 2	254.4±11.0
	Dry 3	241.6±15.8
	Dry 4	161.3±5.1
	Medium Dry 1	294.2±1.7
	Medium Dry 2	147.1±3.2
	Medium Dry 3	163.7±19.0
	Medium Dry 4	78.5±1.5
	Medium Sweet 1	108.0±1.7
	Medium Sweet 2	180.5±3.2
	Medium Sweet 3	347.5±19.0
	Medium Sweet 4	63.8±2.1
	Medium Sweet 5	299.6±0.6
	Sweet 1	227.3±20.7
	Sweet 2	121.6±7.5
	Sweet 3	98.4±1.8
	Sweet 4	393.6±24.5
	Sweet 5	516.1±16.7
	Sweet 6	401.0±21.0
